# Individual- and system-level determinants of breastfeeding in a low-resource setting

**DOI:** 10.3389/fpubh.2024.1471252

**Published:** 2024-11-06

**Authors:** Miranda G. Loutet

**Affiliations:** Dalla Lana School of Public Health, University of Toronto, Toronto, ON, Canada

**Keywords:** breastfeeding, Bangladesh, low- and middle-income country, public health, individual-level determinants, system-level determinants

## Abstract

The benefits of breastfeeding are widely established and therefore the World Health Organization recommends that every child be exclusively breastfed for the first 6 months of life and continue breastfeeding up to 2 years of age or beyond. However, the rate of exclusive breastfeeding is low globally and has declined in Bangladesh in recent years. In this review, Bangladesh is used as an example to demonstrate the complex individual- and system-level determinants of breastfeeding in a low-resource setting. Mothers face barriers to breastfeeding within the context of marketing by commercial milk formula companies, limited safe alternatives to breastfeeding directly from the breast, and insufficient resources to support breastfeeding in the hospital, community, and workplace setting. Future research and implementation science is required to investigate the overlapping effects between breastfeeding and the high antibiotic use and Caesarean section rates in Bangladesh, along with public health efforts to promote breastfeeding based on robust evidence.

## Introduction

The World Health Organization (WHO) recommends every child be exclusively breastfed for the first 6 months and continue to breastfeed up to two years of age or beyond, while introducing complementary foods starting at 6 months. However, rates of exclusive breastfeeding to 6 months of age remain low globally, with only 48% of infants ever exclusively breastfed below 6 months of age in 2022 (1). In Bangladesh, 53% of infants below 6 months of age were exclusively breastfed based on the 2022 Demographic and Health Survey (DHS) and the rate of exclusive breastfeeding has remained relatively stable between 53 and 65% over the past decade (2, 3). However, rates of infants exclusively breastfed up to 6 months of age are likely even lower than these estimates because the DHS collects data on infant feeding practices using a single (cross-sectional) 24-hour recall from caregivers of infants below 6 months of age, which does not capture the dynamic nature of early-infant feeding with periods of time when an infant may be breastfeeding, formula-feeding, or being given other liquids that is common in low- and middle-income countries (LMICs) (4). An observational study conducted in 8 LMICs showed that using the WHO indicator (which uses DHS data) the proportion of infants 0–5. 9 months who exclusively breastfed was 71% among Bangladeshi infants compared to only 10% of infants were exclusively breastfed up to 6 months of age (i.e., met the WHO recommendation) using longitudinally collected 24-hour recalls of infant feeding practices biweekly from shortly after birth to 6 months of age (4).

It is widely established that breast milk contains multiple components that support infant’s growth and development of their immune system and gut microbiome (5–7). Exclusive breastfeeding in particular is important in LMICs because exclusive breastfeeding prevents infections through consumption of contaminated non-human milk feeds and suboptimal breastfeeding causes an estimated 600,000 annual child deaths from pneumonia and diarrhea alone (8, 9).

Recent evidence suggests that breastfeeding may mitigate the effect of antibiotics on the infant gut microbiome, and thereby confer a protective effect against antibiotic-associated risk of asthma (10, 11). This is especially important in a context such as Bangladesh where antibiotic-use and the prevalence of atopic dermatitis and asthma among infants are high (12–15). In a study of longitudinal birth cohorts from 8 LMICs, the average antimicrobial courses per child-year up to 2 years of age was double in the Dhaka, Bangladesh birth cohort compared to the global cohort, 10.3 and 4.9, respectively (13). By 6 months of age, over 98% of infants in the Dhaka birth cohort had received antibiotics. Furthermore, a study of inpatient antimicrobial prescribing among infants 0–12 months in Dhaka found that antimicrobials were prescribed in 73% of admissions (14). The study also assessed if the use of antimicrobials was appropriate based on the use of ‘access’ (should be widely available, affordable and quality assured), ‘watch’ (high resistance potential and should be limited) and ‘reserve’ (last resort in highly specific patients to preserve effectiveness) antimicrobials. Overall, 58% of antibiotics that were prescribed were classified as ‘access’, 38% as ‘watch’ and 1% as ‘reserve; with ‘watch’ antimicrobials used in 26% of neonatal sepsis cases and 76% of lower respiratory tract infection admissions. Antimicrobials were also used in 51% of gastroenteritis and 28% of neonatal jaundice admissions, which were likely viral illnesses or conditions for which antibiotics do not usually confer benefit. Finally, among infants aged 2–6 months brought to a hospital in Dhaka for management of diarrhoeal illnesses, 52% had received antibiotics before hospital admission to treat the diarrhoeal disease for which they were seeking medical attention, possibly due to the fact that medicines are available from diversified sources and antimicrobials may be obtained without physician prescription (15). However, this phenomenon is not unique to Bangladesh, as a study of over 3,000 hospitalized infants less than 60 days of age from 11 countries (mainly Asia and Africa), showed that 98% received antibiotics, with the majority considered ‘watch’ (66%) (16).

This highlights the need for research efforts to focus on promotion and support for breastfeeding based on robust evidence of associations with modifiable risk factors. Although there are known biological determinants of breastfeeding such as gestational age, birthweight, and other maternal and infant health conditions, this paper will focus on structural—both at the individual- and system-level—determinants of breastfeeding in Bangladesh and the main settings that are influenced by these determinants: health systems, communities, workplaces, governments, and commercial. These determinants and settings are the focus of this paper due to their modifiable—although complex—nature and potential for intervention. These challenges are present in many LMICs (17), but Bangladesh is presented in this paper as an example.

## Hospital-level determinants

Bangladesh has the highest rate of live births by Caesarean section (C-section) within health institutions among countries where less than 60% of births were institutional births (18) and in 2022, 45% of live births in the 2 years preceding the DHS-2022 were delivered via C-section (3). C-sections have been shown to be associated with lower rates of mother-infant skin-to-skin contact immediately after birth (19–22), which further significantly reduces rates of breastfeeding initiation (23, 24) and exclusive breastfeeding at 3 and 6 months (25). Although C-sections provide an important life-saving intervention to mothers and newborns, they also result in an increased risk of complications, with 62% of mothers experiencing severe acute morbidity resulting from a C-section in LMICs compared to less than 1% in HICs, and long-term consequences for mother and child (26–30). For instance, being born vaginally has been associated with a lower risk of atopic disease in infants compared to those born by C-section (31). Therefore, C-sections should only be used when medically indicated (32).

Rates of C-section use have increased greatly in LMICs, which may reflect an increase in the number of women giving birth in medical institutions, an increase in access to C-sections, but also possibly a change in the distribution of reasons for C-sections. There is a dearth of evidence on the reasons for C-sections in Bangladesh, but risk factor analyses using the Bangladesh DHS from 2017 to 2018 reported that C-section delivery was higher among women with higher education, from wealthier households, urban areas, and those with access to media (33, 34). Looking to other LMICs, a study among nulliparous women in Argentina found that mode of delivery preferences were most strongly influenced by a doctor or midwife, and that sociodemographic factors such as socioeconomic status and age played a strong role in determining the extent of their influence (35).

Pregnant people and new mothers in Bangladesh receive breastfeeding support while attending health facilities during antenatal care and within 2 days of giving birth; however, access is far from universal (36–38). In 2001, WHO and UNICEF launched the Baby-Friendly Hospital Initiative (BFHI) so that hospitals could be accredited for their commitment to protect, promote, and support breastfeeding (39). An influential cluster-randomized trial called Probit, conducted in Belarus from 1996 to 1998, modelled the intervention on the BFHI and found a large significant effect on any breastfeeding at 12 months (20% vs. 11% in controls) and exclusive breastfeeding at 3-months (43 vs. 6% in controls) and 6-months (8% vs. 0.6% in controls) (40). Even though Bangladesh has made strides in implementing BFHI, the latest report in 2016 showed only 1.5% of hospitals had the designation and reported challenges with training and funding (41, 42). In 2012, the Bangladesh Ministry of Health and Family Welfare established a partnership with the Bangladesh Breastfeeding Foundation (BBF), a non-governmental organization (NGO), to provide technical support to the BFHI programme through capacity building, training, and monitoring (43, 44).

A promising approach to improve breastfeeding support in facilities is increasing the midwife workforce, which is a critical part of Bangladesh’s sexual, reproductive, maternal, newborn, and adolescent health (SRMNAH) strategy (45). In 2008, Bangladesh upgraded its midwifery workforce to meet global standards, with 8,000 registered midwives deployed to government hospitals, NGOs in humanitarian settings, and in private facilities (46). A 2019 qualitative evaluation of interviews with maternity ward staff from government sub-district hospitals in Bangladesh where midwives had been deployed compared to hospitals without midwives indicated that midwives improved quality of care through interventions including skin-to-skin contact, breastfeeding, and obstetric emergency and post-partum management (47). By 2023, midwives in public hospitals were in charge of labour wards and attended up to 85% of births, which led to significant increases in positive birth practices, including use of antenatal card and partograph, upright birth positioning, and mother-infant skin-to-skin contact (46, 48).

## Community-level determinants

Bangladeshi mothers receive support for breastfeeding within the community from a variety of formal (i.e., through funded programmes) and informal (i.e, family and other community members) sources. Formal programmes have been informed by evidence; however, sustainability, standardization and scale-up remain a challenge nationally (49, 50). For instance, a 2-year follow-up to Alive & Thrive’s intensive community-based infant and young child feeding (IYCF) intervention implemented by the local NGO BRAC in rural Bangladesh showed that exposure to some aspects of the intervention had decreased significantly after external funding support from the initial donor agency ended (51). Although there was a sustained impact on early initiation and exclusive breastfeeding along with other IYCF practices and knowledge in areas that received the intervention compared to those that did not, there had been no major scale-up of the intervention to areas that did not receive the initial intervention. Another example is the BBF-led program to implement Mother Support Groups to improve maternal and child nutrition at the community level (52), which is supported with evidence from studies using peer support for lactating women in Bangladesh to effectively improve exclusive breastfeeding to 6 months of age in both urban (53) and rural (54) settings, and specifically among factory workers (55).

Similar to hospital-based breastfeeding support, the increase in the midwife workforce in Bangladesh poses an opportunity for community-level support because midwives can work in the community providing information and breastfeeding support. This is currently being done by midwife students, but overall qualitative evidence from midwives shows that the midwifery centre care model is inaccessible to communities due to challenges of traditional practices and the need for wider acceptance of the midwifery-led care model (56). A study using the Lives Saved Tool modelled the effect of scaling-up the coverage of health interventions delivered by professional midwives on a number of maternal and health outcomes including mortality and exclusive breastfeeding and found that increasing the target coverage of breastfeeding promotion as part of midwives scope of work would have a significant effect on the proportion of children aged 1–5 months who are exclusively breastfed (an increase from 37 to 55% in the lowest human development index (HDI) countries) (57). To note, Bangladesh was categorized as a low-to-medium HDI country and data on exclusive breastfeeding was not presented in the paper on all country groups, but substantial scale-up of midwife interventions had the greatest impact on maternal and neonatal death in the low-to-medium HDI countries, which account for a large proportion of the worlds populations and have high baseline mortality rates.

In Bangladesh, social factors within the community such as family structure and gender norms also impact the breastfeeding journey. A systematic review of facilitators and barriers to early initiation of breastfeeding in South Asia reported that influence of a mother-in-law on maternal and newborn care and lack of the mother’s involvement in decision-making were barriers to early initiation of breastfeeding in Bangladesh (58). Qualitative evidence from interviews with Bangladeshi mothers also identified pressure from older adult family members to feed their infants commercial milk formula (CMF), water and semolina and to prioritise other household chores as barriers to exclusive breastfeeding (59, 60). There is some qualitative evidence that mothers in Bangladesh consider the sex of the infant when deciding to continue to breastfeed due to differences in infant behaviour (59); however, this is not supported by large quantitative national surveys that show no difference in breastfeeding practices by infant sex (3, 61, 62). Importantly, in a study of parents with a child 0–6 months of age from a nationally-representative sample in Bangladesh, fathers’ knowledge about exclusive breastfeeding and support to mothers to practice exclusive breastfeeding had a significant positive impact on maternal exclusive breastfeeding knowledge and attitude (63). Consideration of these cultural and social factors is essential to designing breastfeeding promotion interventions because for instance, male engagement does not consistently improve rates of exclusive breastfeeding across all LMICs with different gender norms (64). In Bangladesh, Alive & Thrive’s intensive IYCF intervention that successfully increased early initiation and exclusive breastfeeding included mass media campaigns targeted at mothers, fathers, and community leaders (65, 66).

## Female labour force participation

Returning to work is one of the top reasons for not breastfeeding reported by new mothers in LMICs (67). In LMICs, where a high proportion of work is in the informal sector, underpaid and unprotected, many women face the challenge of competing priorities between the time required to breastfeed and other care and income-earning responsibilities (68). Workplace support for breastfeeding includes low-cost strategies that are cost-effective, especially given the high value of breastfeeding such that in 2020 the global monetary value of women’s milk production among infants 0–36 months was approximately $US 3.6 trillion (68–70). To create an enabling workplace environment for breastfeeding, a study in South Africa developed a comprehensive practice model based on critical review of the literature, mixed-methods data collection and consensus from experts, which was centered on time, space and support inputs by the employer, measurable outputs, and short to long-term outcomes (71). The methodology, and possibly even the practice model itself, has the potential to be applicable in other LMIC settings.

In Bangladesh, female labour force participation has been increasing (26% in 2003 to 36% in 2016) (72) and 85% of the estimated 4 million garment factory workers are women of reproductive age (73). However, these women lack information and support to continue breastfeeding when they return to work. In a pooled analysis of the Bangladesh DHS from 2011 to 2018, employed mothers had 24% lower odds of any exclusive breastfeeding between 0–5.9 months (adjusted odds ratio = 0.76, 95%CI: 0.59–0.96) (74). A survey in two factories in Dhaka, Bangladesh found that only 17% of female factory workers with infants aged below 2 years exclusively breastfed their infants up to 6 months of age (75). Furthermore, qualitative research among garment factory workers who were mothers of 0–12 month old infants showed that mothers introduce CMF as early as 2 months postpartum because they had very little knowledge about the use of expressed breast milk, did not have access to breast pumps or refrigeration at work or home, and were concerned about pathogenic contamination of expressed human milk due to this lack of refrigeration (76). Additionally, these mothers faced barriers to breastfeeding due to excessive workload without scheduled breaks, inadequate child-care facilities at work, and caregivers at home who, understandably, were unable to bring the infants to the factories for feeding.

There have been a number of promising interventions to support Bangladeshi mothers returning to work. In a two-group longitudinal mixed-methods study comparing the effectiveness of a home-based peer support programme from 6 months of pregnancy until 6 months postpartum among pregnant and lactating factory workers compared to their unemployed female neighbors in Bangladesh, exclusive breastfeeding at 6 months was high among both groups (86% in employed group and 95% in unemployed group) (54). Peer counsellors in that study educated mothers on safe expression, storage and feeding of breast milk, which enabled employed mothers to feed infants their breast milk exclusively when returning to work and encouraged family members to trust and help throughout the process. However, employed mothers still described challenges with finding the time and space in the workplace to express their breastmilk. Recently, there has been more organizational commitment to support working mothers in Bangladesh. In 2016, UNICEF and the Bangladeshi government including the Ministry of Health and Family Welfare and Ministry of Labour and Employment convened a task force to improve maternity protection and infant and child care in Bangladeshi businesses (75, 77). UNICEF also supported the establishment of an advocacy programme to strengthen maternity rights and protect breastfeeding in the workplace called Mothers@Work. This initiative partnered with two factories in Bangladesh to act as pilot projects to support breastfeeding in the workplace (78). Most recently, the UNICEF-led Mothers@Work initiative partnered with Bangladeshi factory associations to support factories to provide breastfeeding spaces and breaks, childcare facilities, paid maternity leave, and a safe work environment for working mothers and pregnant women (79).

## Commercial milk formula use

Overall, due to the high expense of CMF, breastfeeding is more prevalent in LMICs than high-income countries (5), and within LMICs, CMF-use is positively associated with household wealth (80). In Bangladesh, rates of CMF use are highest among the wealthiest households (17.4% from DHS-2014), but rates are still high among the poorest (9.5% from DHS-2014) (81) for which the risk and consequences from unsterile bottles and/or contaminated water supply is greatest (5, 9). The rate of CMF use is also increasing in Bangladesh, with 22% of infants aged between 0 and 5 months received mixed milk feeding (breast milk and CMF and/or fresh, packaged, or powdered animal milk) based on the DHS-2022, which was highest among the wealthiest households (24%) and lower among households in the lowest wealth quintile (13%) (3). Furthermore, a recent report by WHO and UNICEF from 2022 showed that 27% of women surveyed in Bangladesh were exposed to CMF marketing and 57% received recommendations from health professionals to use CMF products (82).

In 1981, the WHO developed the International Code for the Marketing of Breast-milk Substitutes and called on countries to enact individual laws and regulations to limit the marketing methods for CMF and related products (83). However, as of 2023, only 32 countries have legal measures substantially aligned to the Code and there has been an increase in advertising expenditure by CMF manufacturers by 164% in the past decade (84, 85). Violations of the WHO International Code are a global challenge and there is a substantial gap in multilevel and multicomponent interventions to address them (17, 68, 84–86). LMICs that have made progress against CMF markets, such as the Philippines, have done so through political commitment including an official database of reported violations of the Code and coalitions to resist the CMF industry (17, 68, 85). Although the Bangladesh Breastmilk Substitutes Act was developed and adopted by the Bangladeshi Parliament in 2017, the rates of exposure to CMF marketing and use of CMF highlight the need for robust implementation, stronger enforcement, and monitoring of the Act to protect families at all socioeconomic levels from unsubstantiated claims about CMF (87).

## Conclusion

Factors affecting breastfeeding practices are often structural, socioeconomic, cultural, biological, and medical; and therefore, breastfeeding promotion requires multifaceted public health efforts to target these factors (17, 68, 69). Breastfeeding rates are impacted by all of these factors in Bangladesh within the context of marketing by CMF companies, limited safe alternatives to breastfeeding directly from the breast, and insufficient resources to support breastfeeding in the hospital, community, and workplace setting. The WHO cautions against the use of feeding bottles and breast-milk substitutes, especially in LMICs, due to the high risk of introducing contamination that can lead to life-threatening infections in young infants (88), so the fears expressed by Bangladeshi mothers of returning to work and risk among low-income households (81) are warranted. Encouragingly, the rate of exclusive breastfeeding is higher in Bangladesh compared to other countries worldwide. The overlapping effects of high antibiotic use and C-section rates may have even larger negative effects on infant health outcomes such as atopic disease if it were not for the protective effect of breastfeeding in this context; however, more research on these interrelated factors is required.
